# ProSTAGE: Predicting Effects of Mutations on Protein Stability by Using Protein Embeddings and Graph Convolutional Networks

**DOI:** 10.1021/acs.jcim.3c01697

**Published:** 2024-01-02

**Authors:** Gen Li, Sijie Yao, Long Fan

**Affiliations:** Production and R&D Center I of LSS, GenScript (Shanghai) Biotech Co., Ltd., Shanghai 200131, China

## Abstract

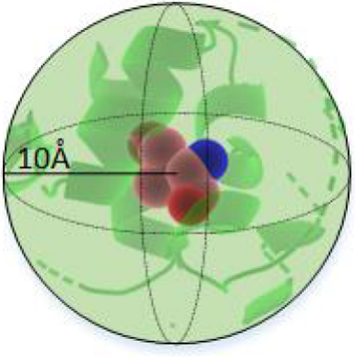

Protein thermodynamic stability is essential to clarify the relationships among structure, function, and interaction. Therefore, developing a faster and more accurate method to predict the impact of the mutations on protein stability is helpful for protein design and understanding the phenotypic variation. Recent studies have shown that protein embedding will be particularly powerful at modeling sequence information with context dependence, such as subcellular localization, variant effect, and secondary structure prediction. Herein, we introduce a novel method, ProSTAGE, which is a deep learning method that fuses structure and sequence embedding to predict protein stability changes upon single point mutations. Our model combines graph-based techniques and language models to predict stability changes. Moreover, ProSTAGE is trained on a larger data set, which is almost twice as large as the most used S2648 data set. It consistently outperforms all existing state-of-the-art methods on mutation-affected problems as benchmarked on several independent data sets. The protein embedding as the prediction input achieves better results than the previous results, which shows the potential of protein language models in predicting the effect of mutations on proteins. ProSTAGE is implemented as a user-friendly web server.

## Introduction

Thermodynamic stability is one of the most fundamental properties of protein that significantly influences protein structure, function, expression, and solubility.^1^ It is well-known in the clinics that mutations may reduce the thermodynamic stability of proteins. Such mutations can result in misfolding of gene products, numerous genetic disorders, cancers, and neurodegenerative diseases.^2^ Therefore, assessing the effect of mutations on the protein thermodynamic stability (ΔΔ*G*) is crucial in the development of a wide range of biotechnology products, including protein-based therapeutics, biocatalysts, and other applications.^3^ While experimental measurements are preferred, conducting a thorough study on a protein is impractical. Developing a computational method is a crucial step to design customized proteins for protein engineering, personalized medicine, and precision diagnostics.^4^

In the past 30 years, the development of protein stability prediction has become a highly active research area, and dozens of methods have been developed.^5^ Most of these methods are based on artificial intelligence, which typically use sequence, structure, physical force field, and evolutionary information^4,6^ as features to predict the impact of mutations. The published AlphaFold overcomes the limitation of structure-based methods that cannot be used due to the lack of structure. On the other hand, AlphaFold^7^ demonstrates the power of deep learning, especially Natural Language Processing (NLP) on modeling sequence information with context dependence.^8^

The recent revolutionary development of NLP has influenced protein research work, and several studies have applied the concept of language models to protein sequences, such as UniRep,^9^ EMS-1b,^10^ TAPE,^11^ and ProtTrans.^12^ Their studies show that protein sequence embedding (the vector representation output by pretrained model) is capable of accurately predicting subcellular localization and SCOP. These pretrained models provide us with a unique insight into the language in form of embeddings, which are found to be effective in solving various downstream tasks and significantly improved upon the earlier supervised machine learning based methods trained on task-specific smaller data sets.

However, the majority of existing methods continue to rely on traditional shallow machine learning techniques. The reason is that deep learning methods demand a substantial amount of input data for effective training.^13^ Since standard training data sets, such as S2648 or Q3421,^14^ typically contain only a few thousand entries, they are considered to be insufficiently large to support the application of these advanced techniques.

In this respect, we developed a novel approach: ProSTAGE, which is a deep learning method that fuses structure and sequence embedding to predict protein stability changes upon single mutations. Our model combines graph-based techniques and language models to predict stability changes. The advantage of using the language model-based feature vectors is that it does not require domain knowledge to encode the sequences.^15^ For the graph, we propose a spatial node feature to capture the residue interaction properties near mutations and use the protein sequence embedding layer of the protein language pretrained model as the spatial node features input to Graph Convolutional Networks (GCN). Also, to help relieve the bottleneck of model performance limited by the “data shortage”, ProSTAGE trained on the training set we collected from multiple sources, which is twice as large as the most used S2648 data set. It is shown to consistently outperform all existing state-of-the-art methods on mutation-affected regression problems as benchmarked on several independent data sets. ProSTAGE is implemented as a user-friendly web server.

## Materials and Methods

### Data Set Collection

#### Training Data Sets

We compiled a new curated data set screened from the four newly published databases: MPTherm,^16^ ProthermDB,^17^ ThermoMutDB,^18^ and FireProtDB.^19^ These mutations with known ΔΔ*G* satisfied the following rules: (1) single point mutations with ΔΔ*G* and (2) nonredundant data. 4335 mutations decrease stability (ΔΔ*G* ≤ 0) and 1317 mutations increase stability (ΔΔ*G* > 0). To balance the stabilizing mutations and destabilizing mutations, we did the same way as other methods to satisfy the antisymmetric property.^20−22^ More specifically, if protein B is a mutant of protein A, we have ΔΔ*G*(A/B) = −ΔΔ*G*(B/A). This leads to our final training set S11304 (11,304 mutations across 318 proteins), which is the largest training set ever used for protein stability prediction.

#### S669

A strict and widely used blind test set. It consists of 669 single mutations that complied by Pancotti et al.^5^ This data set contains experimental thermodynamic information (ΔΔ*G*) for single mutations.

#### Tm262

A blind test set contains only the *T*_m_ experimental values. Since our training data set contains almost all experimental ΔΔ*G* data available now, we additionally designed a blind test set to fairly test the performance differences of current advanced methods. It consists of 262 single mutations with experimental Δ*T*_m_ from the same source as that for the training data set. In addition to the similar training set filtering criteria, the following rules are also used: (1) the experimental values only have Δ*T*_m_, (2) |Δ*T*_m_| ≥ 10 °C.

#### PTEN and TPMT

The third blind test set is a deep mutational scanning (DMS) data set from the CAGI5 challenge, including the phosphatase and tensin homologue (PTEN) and thiopurine *S*-methyl transferase (TPMT) proteins, a total of 7363 mutations for the PTEN (3736) and TPMT (3627) proteins. It was downloaded from https://genomeinterpretation.org/content/predict-effect-missense-mutations-pten-and-tpmt-protein-stability. The detailed information about all the data sets can be found in Tables S1–S4.

### Graph Convolutional Network Architecture

Given a graph, *G* = (*V*, *E*), where *V* is the set of *K* nodes and *E* are edges. The input of GCN is

1. Node features *X*, *X* has dimension *N* × *F*^0^, where *N* is the number of nodes and *F*^0^ is the number of features for each node.
2. The adjacency matrix *A* of the graph, the dimension of *A* is *N × N*.

In the protein, the nodes represent the amino acids of which proteins are made up, and the interactions between residues make up the adjacency matrix. The input of the model is the graph represented by its adjacency and node features matrices. There are three GCN layers, which take both the spatial adjacency matrix and the embeddings from the previous layer and output the embeddings in the next layer. Each graph convolutional layer had 64 units. The embeddings of three graph convolutional layers are concatenated into one matrix. This is then followed by a pooling layer, and additional knowledge-based features are added to this layer. After that, three fully connected layers are used for mapping the embeddings from the previous layer which outputs the embeddings in the next layer, and the final layer outputs a value for predicting ΔΔ*G*.

In this work, we built GCN model using the class of dgl.nn.pytorch.conv.GraphConv in DGL (version 1.1.0),^23,24^ also we used pseudocode to describe the workflows of our model development, which can be found in the Supporting Information.

### Node Features

Graph-based protein node features usually adopt one hot spot to encode the characteristics of each node, which can be physicochemical properties or evolutionary information. In this work, we applied the protein sequence embeddings generated by the ProtT5-XL-Uniref50 pretrained model as node features. ProtT5-XL-Uniref50 pretrained model was developed by Elnaggar et al.,^12^ which was trained on 450 M protein sequences by using the T5 architecture with 3B parameters. It is a transformer-based architecture that adopts an encoder-decoder and randomly masks 15% of the amino acids in the protein sequence corpus. The learning rate is 0.01, local batch size is 8 and global batch size is 2048, dropout rate is 0.1, and the AdaFactor optimizer is used for the model optimization. The number of hidden layers is 1024, and the number of layers is 24 with 32 attention heads. It achieves state-of-the-art results in multiple downstream tasks compared with other popular protein language models.^25^ The traditional way to obtain the node features is based on the sequence, and selected several amino acids from both the left and right of the mutation site as nodes.^26^ Obviously, it is more reasonable to construct nodes through space, because mutations change the surrounding interactions. One mutation is represented as the concatenating of wild sequence embedding and that of the mutant sequence. This means, the node feature is a matrix of *N* × 2048, where *N* is the set of residues less than 10 Å away from the mutation site and *F*^0^ = 2048 is the twice size of hidden layers. We denote the matrix as mutational embedding, which represents the mutational effect on the whole sequence.

### Spatial Adjacency Matrix (SAM)

Adjacency matrix indicates whether pairs of vertices are adjacent to each other or not in the graph. This theory is based on the protein structure as a network, in which each amino acid is directly connected according to a certain relationship; the connection between each node and other nodes is the interaction between an amino acid and other surrounding amino acids. For geometric models, we defined the Cα atom of each residue as a node, and edges were drawn between nodes if they were within 10 Å from each other. Mutational embedding corresponding to each residue was then assigned to the respective node on the protein graph.

### Additional Knowledge-Based Features (AKB)

Moreover, a set of additional knowledge regarding the environmental characteristics of the wild-type residue (e.g., relative solvent accessibility, conservation score, and secondary structure) was added to fully connected layers. The detailed information is described in Supporting Information.

## Results

### Designing a Novel Graph Convolutional Network Framework

**Figure 1:**
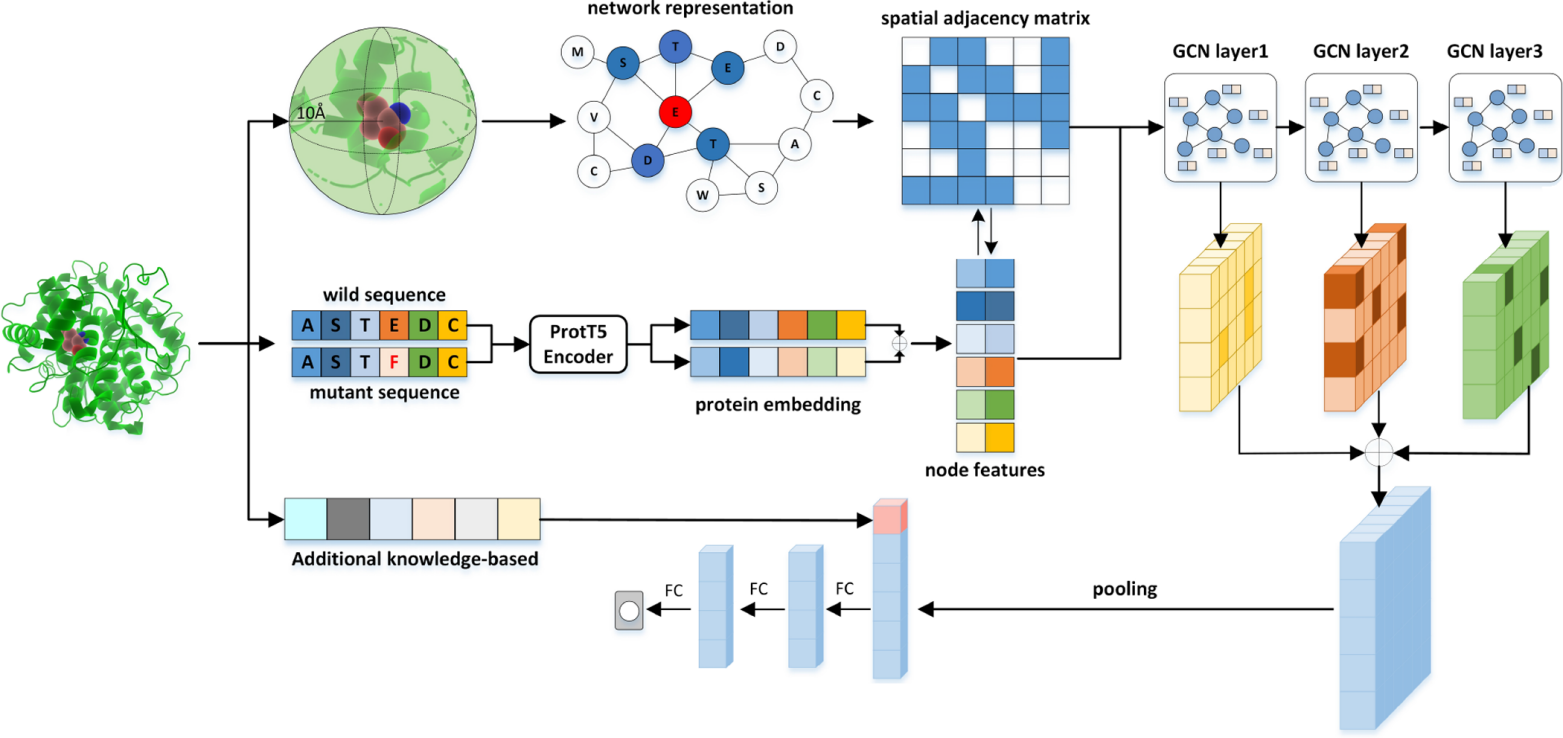
Overview of the model architectures. ProSTAGE extracts two parts of information from the protein 3D structure: interaction network and sequence embedding. The protein embedding was taken from ProtT5-XL-Uniref50, each square represented a 1024-dimensional feature, and then a 2048-dimensional feature was obtained after concatenating wild and mutant. The interaction network is composed of those residues that were less than 10 Å away from the mutation site. There are three graph convolutional layers, which take both the spatial adjacency matrix and the embeddings from the previous layer, and outputs the embeddings in the next layer. The embeddings of three graph convolutional layers are concatenated as one matrix. This is then followed by a pooling layer, and additional knowledge-based features are added to this layer. We then use three fully connected layers for computing the embedding from the pooled representation. Finally, the ΔΔ*G* value is predicted by the GCN model.

The deep learning approach ProSTAGE was developed by combining GCN for structures and protein embedding for sequences. The GCN architectures of ProSTAGE used the protein embeddings, spatial adjacency matrix, and additional knowledge-based features as the input to train the model. GCN was used to capture short-range residue interactions of mutation sites, while a language model was used to represent long-range protein sequence information. We utilized S11304 as the training set and others as the test set for evaluating the performance. ProSTAGE adopted a loss function here, which includes the mean square error (MSE) between predicted and experimental values. To avoid overfitting, we use an early stopping criterion with patience = 5. The model architecture is shown in Figure 1.

**Figure 2:**
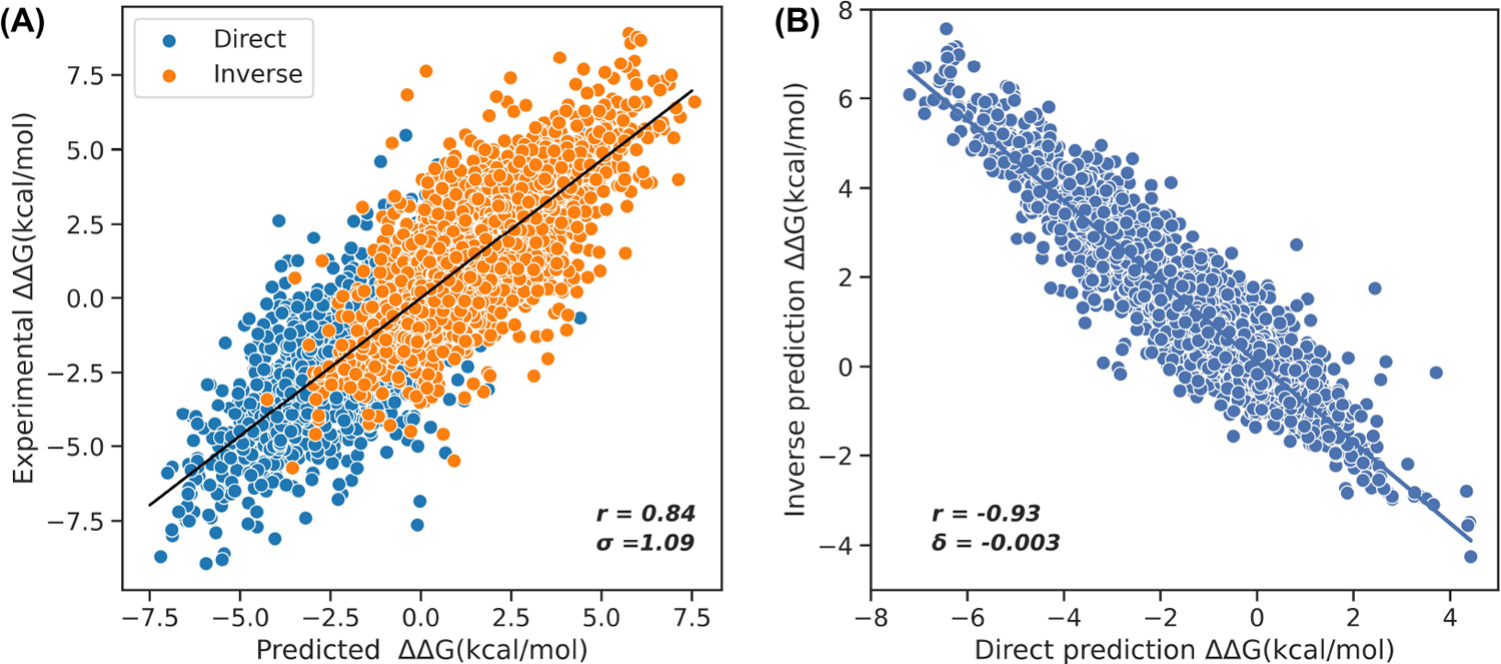
5-fold cross-validation result of the training data set. (A) Overall Pearson correlation coefficient (PCC, *r*) and RMSE (σ) of direct (orange) and inverse (blue) predictions. (B) *r* and bias (δ) between direct and inverse predictions.

We trained ProSTAGE on the S11304 data set as described in the Methods section. The 5-fold cross-validation performance of the model on 20% of the training set is shown in Figure 2. The Pearson correlation coefficient (PCC) is 0.84 and RMSE is 1.09 kcal/mol. The antisymmetry property is satisfied perfectly, with PCC between direct and inverse mutations being −0.93 and bias (δ) of just −0.003 kcal/mol. Also, leave-one-protein-out cross validation (LOPOCV) is performed to further test the model performance with the PCC and RMSE values of 0.75 and 1.34 kcal/mol, respectively (Figure S1).

### Comparison of ProSTAGE Performance with Other Methods

To assess the ability of the ProSTAGE to predict the effect of mutations on protein stability, we designed an extensive series of comparative experiments with other recently state-of-the-art methods, including PROST,^27^ PremPS,^21^ ACDC-NN,^22^ DDGun,^28^ ThermoNet,^29^ Dynamut,^30^ and Rosetta^31^ (only methods that performed in the top 7 in the Pancotti et al. were selected^5^). We employed four universal nonredundant blind test sets of S669, Tm262, PTEN, and TPMT to further benchmark it.

### S669 Data Set

Tools that predict the effect of single mutations on protein thermostability are commonly benchmarked on S669,^5^ which is one of the newly balanced and strict blind data sets. Most recently published tools for predicting protein stability are tested on this data set for a fair comparison. We used two ways for fair comparison: (1) removed a part of the mutations that are homologous to the S669 data set from our method’s training set and (2) used S2648 as the training set. The results of the comparison are listed in Table 1.

**Table 1:** Comparison of ProSTAGE with Existing State-of-the-Art Predictors on the S669 Data Set^a^

method	total			direct			inverse			antisymmetry	
	*r*	RMSE	MAE	*r*	RMSE	MAE	*r*	RMSE	MAE	*r*_d–i_	⟨δ⟩
ProSTAGE	**0.70**	**1.37**	**0.97**	**0.57**	**1.36**	**0.94**	**0.55**	**1.38**	**1.00**	–0.92	0.03
ProSTAGE (S2648)	0.67	1.42	1.03	0.51	1.41	1.03	0.49	1.42	1.03	–0.84	–0.01
PROST	0.64	1.46	1.03	0.47	1.46	1.02	0.47	1.46	1.04	–0.91	–0.02
PremPS	0.62	1.49	1.07	0.41	1.50	1.08	0.42	1.49	1.05	–0.85	0.09
ACDC-NN	0.61	1.50	1.05	0.46	1.49	1.05	0.45	1.50	1.06	–0.98	–0.02
ACDC-NN-Seq	0.59	1.53	1.08	0.42	1.53	1.08	0.42	1.53	1.08	**–1**	**0**
DDGun3D	0.57	1.61	1.13	0.43	1.60	1.11	0.41	1.62	1.14	–0.97	–0.05
DDGun	0.57	1.74	1.25	0.41	1.72	1.25	0.38	1.75	1.25	–0.96	–0.05
ThermoNet	0.51	1.64	1.20	0.39	1.62	1.17	0.38	1.66	1.23	–0.85	–0.05
Dynamut	0.50	1.65	1.21	0.41	1.60	1.19	0.34	1.69	1.24	–0.58	–0.06
Rosetta	0.47	2.69	2.05	0.39	2.70	2.08	0.40	2.68	2.02	–0.72	–0.61

a The δ, RMSE, and MAE values for direct and inverse mutations are expressed in kcal/mol, the Pearson correlation coefficient *r*_d–i_ between the predicted ΔΔ*G* values of direct and inverse mutations, and the bias δ are listed. Results are taken from Pancotti et al.^5^ Best results in bold.

ProSTAGE outperforms all other predictors by reaching a PCC = 0.70, RMSE = 1.37, and MAE = 0.97 kcal/mol on the S669 total data set, and the second-best predictor on the total data set is PROST (PCC = 0.64, RMSE = 1.46, and MAE = 1.03 kcal/mol). On the S669 direct mutation, the trend is similar to the total data set. Our method still achieves the highest performance: PCC = 0.57, the lowest RMSE = 1.36 kcal/mol, and MAE = 0.94 kcal/mol. On the S669 inverse mutation, ProSTAGE attains impressive values (PCC = 0.55, RMSE = 1.38 kcal/mol, and MAE = 1.00 kcal/mol) in comparison to all other predictors (PCC from 0.34 to 0.47, RMSE from 2.68 to 1.46, and MAE from 2.02 to 1.05). These outcomes are likely attributed to the utilization of both direct and inverse mutations during the model training process. Furthermore, we conducted verification on the antisymmetry and calculated an ***r***_d–i_ value between the predicted direct and inverse mutations results. Our antisymmetry results in a value of ***r***_d_–_i_ = −0.92 and ⟨δ⟩ = 0.03. As mentioned in Pancotti et al.,^5^ these predictors (PROST, ACDC-NN-Seq, ACDC-NN, DDGun3D, DDGun, ThermoNet, PremPS) built on antisymmetric perform significantly better than these not-antisymmetric predictors (Dynamut, Rosetta), showing a strong bias toward the destabilizing mutations. It means that the acquired knowledge of thermodynamic properties enables the antisymmetric methods to enhance their performance. Besides, we also trained our GCN model on the most used S2648 data set. The result still outperforms all other predictors by reaching a PCC = 0.67, RMSE = 1.42 kcal/mol, and MAE = 1.03 kcal/mol on the S669 total data set, which illustrates our model architecture and larger data set work together to help our model achieve better performance.

### Ability of ProSTAGE To Identify Stabilizing and Destabilizing Mutations

Due to high-quality data is valuable for the robustness of the model, the previous test set has been incorporated into the training set to enhance the generalization ability of the model in recent methods.^32^ This makes it difficult to compare the different methods fairly. We constructed, cleaned, and manually checked a new benchmark test set Tm262 in this work to enable the model to fairly evaluate on protein stability prediction. Since the *T*_m_ value is not linearly related to ΔΔ*G*, the data set is converted into a classification task. Comparing with other methods, Table 2 indicates the excellent performance of ProSTAGE in predicting stabilizing and destabilizing protein mutations. It achieves AUC of 0.80 and accuracy of 0.81 with a precision and recall of 0.84 and 0.25 respectively. All other methods perform much worse. Also, we take the subset of Tm262 as a new test set, which shares less than 25% sequence identity with training set, named Tm108, for testing the generalization ability of the model. Likewise, ProSTAGE outperforms all other methods. The precision examined the potential of ProSTAGE in protein engineering and identifying pathogenic mutations caused by stability disorder.

**Table 2:** Ability To Identify Stabilizing and Destabilizing Mutations on Tm262 and Tm108 Blind Test Sets^a^

methods	Tm262				Tm108			
	AUC	accuracy	precision	recall	AUC	accuracy	precision	recall
ProSTAGE	0.80	0.81	0.84	0.25	0.71	0.72	0.87	0.32
PremPS	0.77	0.79	0.65	0.24	0.70	0.69	0.68	0.32
ACDC-NN	0.73	0.72	0.42	0.44	0.62	0.56	0.43	0.46
ACDC-NN-SEQ	0.73	0.71	0.42	0.51	0.65	0.60	0.48	0.51
DDGun3D	0.73	0.71	0.43	0.60	0.64	0.60	0.48	0.61
DDGun	0.74	0.73	0.44	0.49	0.65	0.62	0.50	0.59
ThermoNet	0.69	0.61	0.34	0.63	0.60	0.54	0.43	0.66
PROST	0.75	0.77	0.53	0.32	0.68	0.69	0.68	0.37
DDMut	0.69	0.71	0.40	0.40	0.52	0.57	0.40	0.24
Rosseta	0.62	0.59	0.33	0.65	0.48	0.47	0.39	0.66

a Tm108 is a subset of Tm262, which shares less than 25% sequence identity with the training set.

### Performance on DMS Data Set for a Certain Protein Saturation mutation

CAGI5 (Critical Assessment of Genome Interpretation 5 challenge) is the third independent data,^33^ which is composed of 7363 stability scores determined by DMS (Deep mutational scanning) technology,^34^ representing two proteins: Phosphatase and tensin homologue (PTEN) and Thiopurine methyltransferase (TPMT). The sequence identities of PTEN and TPMT share less than 25% with our training set. Table 3 displays a comparison of predictions in terms of PCC obtained using various predictors. ProSTAGE outperforms all other predictors in terms of PCC, with a value of 0.56 and 0.53 for PTEN and TPMT, respectively. This significantly outperformed the previously reported predictors, whose correlations ranged from 0.21 to 0.53 and 0.22 to 0.46 on the PTEN and TPMT data sets, respectively. This suggests that ProSTAGE provides a consistent prediction of different protein stability changes and potential for protein engineering. Meanwhile, it is proved that the predicted structure can be used for the stability prediction without the need of experimental structure, which undoubtedly greatly increases the usability of our method.

**Table 3:** Performance Comparison of ProSTAGE with Other Existing Methods on PTEN and TPMT Data Sets^a^

method	CAGI5 data set	
	PTEN	TPMT
ProtSTAGE	0.56	0.53
PremPS	0.53	0.46
ACDC-NN	0.50	0.38
ACDC-NN-SEQ	0.48	0.37
DDGun3D	0.45	0.39
DDGun	0.37	0.37
ThermoNet	0.29	0.23
PROST	0.43	0.43
DDMut	0.29	0.47
Rosseta	0.21	0.22

a The results presented as PCC.

### Ablation Study of ProSTAGE

Furthermore, we conducted an ablation study of ProSTAGE to analyze the contributions of the model architecture and data enrichment. The baseline model was trained by using T5 embedding as the node features, SAM as the adjacent matrix, and additional knowledge-based features. We tested the performance of our model when T5 node feature was replaced by the amino acid one-hot encoding, AKB was removed, SAM was replaced with sequence features, and training set was replaced with S2648. As shown in Table 4, the SAM and AKB techniques slightly enhance the performance, whereas T5 protein embedding makes a more significant contribution to increase the Pearson correlation coefficient by 0.13 (direct). The result shows that using the protein sequence embedding layer of the protein language pretrained model as the spatial node features input to graph convolutional network produces a better result. Node features with sizes from 10 to 2048 are also tested to verify whether there is an overfitting risk for nodes with a size of 2048, and the results in Table S5 remove this risk and confirm the ability of T5 protein embedding to summarize information. By the way, it proves that the richness of the data is important to the performance of the model when our model uses S2648 as the training set. Overall, the results demonstrate the robustness of ProSTAGE, as it is able to reduce dependence on the availability of evolutionary information, which is not always abundant, such as orphan proteins or rapidly evolving proteins.

**Table 4:** Ablation Study of ProSTAGE^a^

model	total			direct			inverse		
	*r*	RMSE	MAE	*r*	RMSE	MAE	*r*	RMSE	MAE
T5_SAM_AKB (ProSTAGE)	0.70	1.37	0.97	0.57	1.36	0.94	0.55	1.38	1.00
ProSTAGE (S2648)	0.67	1.42	1.03	0.51	1.41	1.03	0.49	1.42	1.03
T5_SAM	0.63	1.47	1.06	0.49	1.47	1.06	0.51	1.47	1.06
T5_sequence_AKB	0.62	1.54	1.11	0.48	1.61	1.18	0.50	1.47	1.05
Onehot_SAM_AKB	0.57	1.59	1.13	0.34	1.60	1.14	0.34	1.58	1.13

a All results are tested on S669. “Sequence” means using the left and right 3 amino acids near the mutation site on the sequence to replace the amino acids near the mutation site in space (SAM) and updating the corresponding adjacent matrix.

### Web Implement

**Figure 3:**
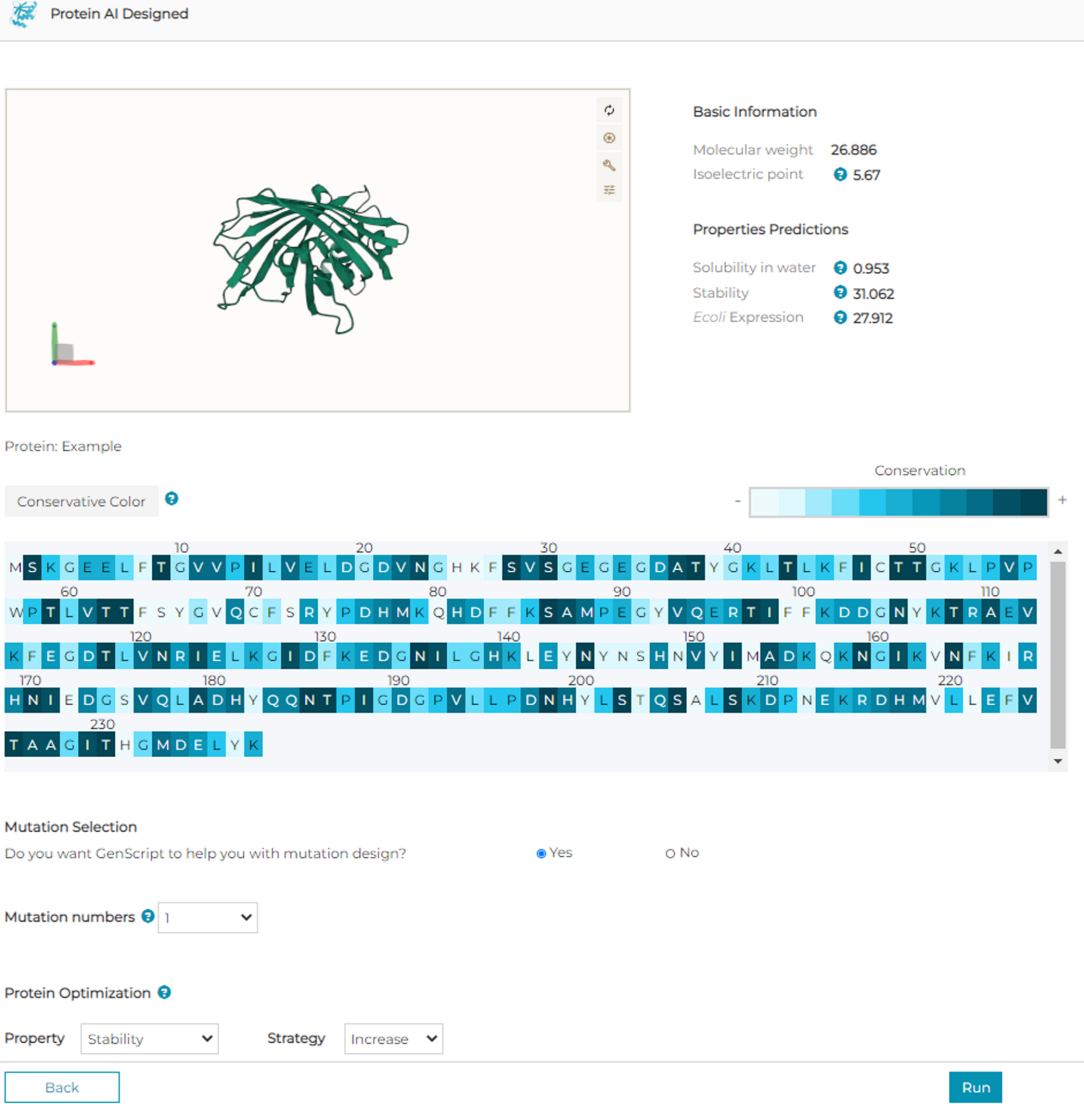
ProSTAGE setup page. The figure depicts the user-friendly interactive 3D view of the structure and sequence view colored by the conservation score. Also, there are force and block functions to customize the result.

We deployed ProSTAGE as an excellent user-friendly web server at https://www.genscript.com/tools/protein-ai-designed. The web server is hosted on a Linux machine running Nginx from Python. There are four steps for easily designing and optimizing the stability of the target enzyme, which are project creation, setup, AI design, and result presentation (Figure 3).

Step 1. User can either paste or upload the structure file, which must comply with the PDB format.

Step 2. Conservation scores help the user better understand proteins and select a suitable mutation site. ProSTAGE provides a user-friendly interactive 3D view of the structure colored by the conservation score.

Step 3. User can select the site that they want to mutate (force) and/or the site that does not allow (block) mutation on a highly visual interface. When all the inputs are set, click submit to start the prediction task.

## Discussion

Protein stability prediction remains a complex and challenging problem. Benefit from the development of deep learning algorithms, the study spot of protein stability prediction has changed from standard shallow machine learning^4,20,21^ to complex deep learning approaches,^22,26^ while traditional training data sets, such as S2648 or Q3421^14^ cannot satisfy the growing training set requirement of deep learning. On the other hand, the emergence of AI-based structure predicted methods eliminate the input requirements between structure-based and sequence-based method for protein stability prediction, allowing people to choose structure-based methods or sequence-based methods according to their demand.

In this regard, we proposed a new predictor and a Web server, ProSTAGE, which is a Graph Convolutional Network utilizing direct and inverse mutations to account for model antisymmetry and integrate protein embedding and spatial adjacency matrix to better capture short-range residue interactions and long-range protein language information. One major advantage of using language model-based feature vectors is that it eliminates the need for domain knowledge to encode the sequences, a previously unexplored avenue in the context of protein stability predictions. The results demonstrate the effectiveness of this approach in predicting the protein stability changes caused by mutations. The Ablation study illustrates that protein embedding can be used to uncover protein language context, which can further improve the predictive accuracy of our model. The ProSTAGE uses a much larger training set (twice as large as S2648) than previously reported approaches to avoid the risk of overfitting and outperforms other predictors on various blind test sets especially in both accuracy and AUC. Moreover, our method exhibits remarkable robustness and performance resilience by attaining high predictive accuracy even when using AlphaFold2 predicted structures as input, thereby dramatically enhancing the scalability of protein stability prediction without compromising on accuracy. We believe ProSTAGE will be a useful tool for various applications such as the finding of key residues, inferring disease-associated mutations, and engineering proteins.

## Data Availability

All the data sets can be found in https://github.com/GenScript-IBDPE/ProSTAGE.

## Supporting Information Available

Data set statistics of training set and blind sets, additional knowledge-based features used in the model, model training details, performance for node features of different sizes, leave-one-protein-out cross-validation result, and pseudocode of ProSTAGE (PDF)
