# Supplementary material for: ProSTAGE: Predicting Effects of Mutations on Protein Stability by Using Protein Embeddings and Graph Convolutional Networks
Source: J Chem Inf Model. 2024 Jan 2;64(2):340–7. doi: 10.1021/acs.jcim.3c01697 (PMC10806799; doi:10.1021/acs.jcim.3c01697)
Supplement: Supplementary file 1 — ci3c01697_si_001.pdf [file ci3c01697_si_001.pdf]

Supporting Information for

ProSTAGE: Predicting Effects of Mutations on Protein Stability by Using Protein Embeddings and Graph Convolutional Networks

Gen Li[a†], Sijie Yao[a†] and Long Fan[a]*

[a]Production and R&D Center I of LSS, GenScript (Shanghai) Biotech Co.,Ltd., Shanghai, 200131, China

[†]These authors contributed equally to this work.

*To whom correspondence should be addressed. Email: leo.fan@genscript.com

Materials and Methods

Table S1. Training set

Dataset	Positive samples ($\Delta\Delta G > 0$)	Negative samples ($\Delta\Delta G \leq 0$)	Proteins Involved
S5652	1317	4335	318

Table S2. S669 blind set

Dataset	Positive samples ($\Delta\Delta G > 0$)	Negative samples ($\Delta\Delta G \leq 0$)	Proteins Involved
S669	168	501	85

Table S3. Tm262 blind set

Dataset	Positive samples ($\Delta T_m \geq 10$℃)	Negative samples ($\Delta T_m \leq -10$℃)	Proteins Involved
Tm262	63	199	83

Table S4. PTEN and TPMT blind set

Dataset	Positive samples (score > 1)	Negative samples (score ≤1)	Proteins Involved
PTEN	839	2897	1
TPMT	896	2731	1

Additional knowledge-based features (AKB)

We use 19 additional features: PSSM score, ΔCS, ΔOMH, $SASA_{pro}$, $SASA_{sol}$, P_{FWY}, P_{RKDE}, P_L, N_{Hydro}, N_{Charg}, ΔConsensus, ΔWolfenden, ΔJanin, $SASA_{pro}/SASA_{sol}$, tco, kappa, alpha, phi and psi as input to the fully connected layer to supplement the representation of the mutation information obtained in the GCN. The detailed information about the first 10 features can be found in Chen et al[1]. ΔConsensus, ΔWolfenden and ΔJanin are taken from Sweet at el.[2], and the angle features come from DSSP[3].

Training detailed

The model utilizes the NVIDIA A100 for training, which is built using the PyTorch deep learning framework and involves extensive hyperparameter tuning. In the 3-layer graph convolutional network (GCN) model, the value of 'norm' is set to both, and both 'weight' and 'bias' are set to true. The output dimension of each GCN layer is 64. In the 2-layer fully connected network, the output dimension of each layer is 64. The activation function is ReLU and dropout rate set as 0.25. The model is trained for 10 epochs using an Adam optimizer with a learning rate 0.001 and a batch size of 128. All hyperparameters are determined through a grid search based on the model’s performance on the validation set.

Result

Table S5. ProSTAGE performance for node features of different sizes.

Node features	Validation set			Test set		
size	*r*	RMSE	MAE	*r*	RMSE	MAE
10	0.79	1.28	0.96	0.75	1.23	0.92
50	0.80	1.26	0.94	0.76	1.22	0.91
100	0.78	1.31	0.99	0.73	1.27	0.96
150	0.76	1.36	1.02	0.71	1.32	1.01
200	0.78	1.30	0.98	0.73	1.28	0.97
500	0.82	1.21	0.89	0.76	1.20	0.88
1000	0.84	1.14	0.84	0.79	1.13	0.84
2048 (ProSTAGE)	**0.84**	**1.13**	**0.83**	**0.80**	**1.13**	**0.83**

We randomly divide the S11304 data set into training/validation/test sets, which are 70%, 20% and 10%, respectively. All experiments are tested on the same data set partitioning. Node features from 10 to 1000 are obtained by using PCA to reduce the dimension of 2048 node features.

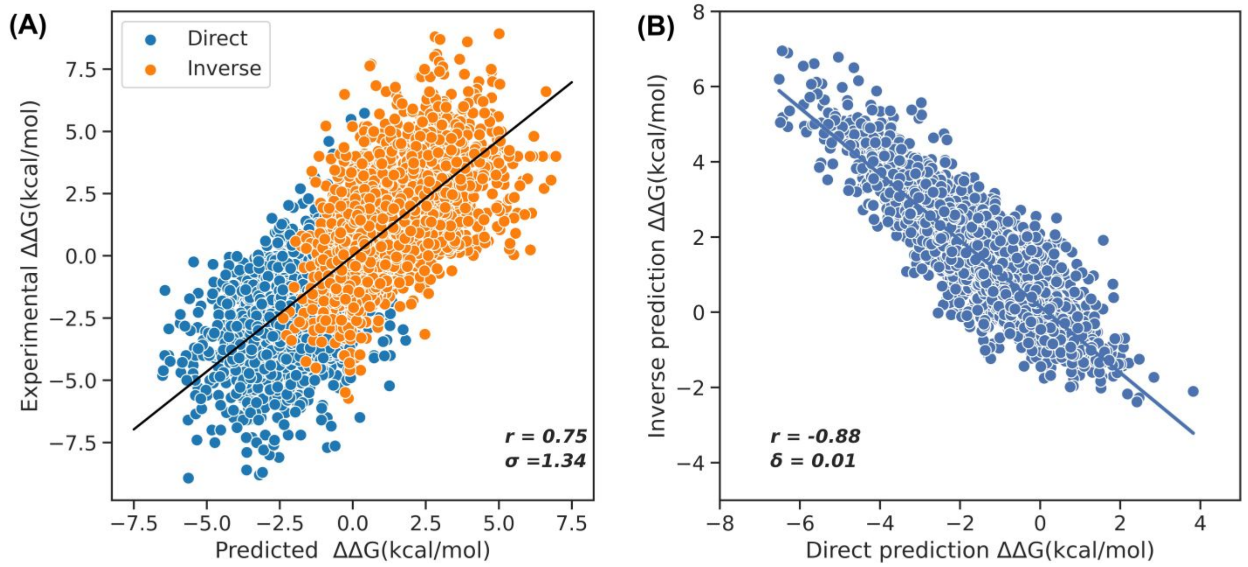

Figure S1. Leave-one-protein-out cross validation result of the training dataset. (**A**) The overall Pearson correlation coefficient (PCC, *r*) and RMSE (σ) of direct (orange) and inverse (blue) predictions. (**B**) The *r* and bias (δ) between direct and inverse predictions.

Pseudocode of ProSTAGE

using ProtT5-XL-Uniref50 model to get sequence embedding as node feature

Function read_node_feature(sequence**)**

```
   node_feature = Embedding_Extraction(sequence)
   return node_feature
End function

# using sequence and structure information to get knowledge based features (AKB)
Function read_knowlegde_based_feature(sequence, structure)
   knowledge_based_embedding = knowledge_based_Embedding(sequence, structure)
   return knowledge_based_embedding
End function

# generate graph architecture
Function graph_generation(structure, cutoff=10, mutation, node_features)
   distance_matrix = write_distance_matrix(structure)
   result_list = []
   for i from 1 to length(distance_matrix) do
      distance = calculate_distance(distance_matrix[i-1], mutation)
      if distance ≤ cutoff then
         result_list.append(i)
   End for
   adjacency_matrix = generate_adjacency_matrix(distance_matrix, result_list)
   graph = write_graph(node_features, adjacency_matrix)
   return graph
End function



# DDGRegressionModel
Function RegressionGraphConvolution(n_conv=3, n_fcn=2, graph)
   node_features = []
   for i = 1 to n_conv:
      node_feature = convolution(graph, graph.node_feature)
```

```
        node_feature = activation(node_feature)
        node_feature = BatchNormalization(node_feature)
        node_feature = Dropout(node_feature)
        node_features.append(h)
    End for
    node_features_mean = Pooling(node_features)
    embedding = concatenate(node_features_mean, knowledge_based_embedding)
    for i = 1 to n_fcn:
        embedding = Linear(embedding)
        embedding = activation(embedding)
        embedding = Dropout(embedding)
    End for
    ddg = Regerssion_linear(embedding)
    return ddg
End function

#training
data = Dataset(graphs, labels)
loss_function = MSELoss
optimizer = Adam
for epoch = 1 to num_epochs:
    for batch in data:
        inputs, targets = batch
        predictions = model(inputs)
        loss = loss_function(predictions, targets)
        optimizer.zero_grad()
        loss.backward()
        optimizer.step()
```

Reference

(1) Chen, Y.; Lu, H.; Zhang, N.; Zhu, Z.; Wang, S.; Li, M. PremPS: Predicting the Impact of Missense Mutations on Protein Stability. *PLoS Comput. Biol.* **2020**, *16*, e1008543. DOI: 10.1371/journal.pcbi.1008543.
(2) Sweet, R. M.; Eisenberg, D. Correlation of Sequence Hydrophobicities Measures Similarity in Three-Dimensional Protein Structure. *J. Mol. Biol.* **1983**, *171*, 479–488. DOI: 10.1016/0022-2836(83)90041-4.
(3) Kabsch, W.; Sander, C. Dictionary of Protein Secondary Structure: Pattern Recognition of Hydrogen-Bonded and Geometrical Features. *Biopolymers* **1983**, *22*, 2577–2637. DOI: 10.1002/bip.360221211.
